# Machine Learning-Based Support for Monitor Unit and Lung Shielding Estimation in Conventional Total Body Irradiation

**DOI:** 10.3390/cancers18111740

**Published:** 2026-05-26

**Authors:** Christian Fiandra, Francesca Romana Giglioli, Elena Gallio, Veronica Richetto, Paola Trevisiol, Matteo Carvutto, Erica Maria Cuffini, Chiara Cavallin, Umberto Ricardi, Mario Levis

**Affiliations:** 1 Department of Oncology, University of Turin, 10126 Turin, Italy; christian.fiandra@unito.it (C.F.); paola.trevisiol@unito.it (P.T.); matteo.carvutto@gmail.com (M.C.); umberto.ricardi@unito.it (U.R.); mario.levis@unito.it (M.L.); 2 Medical Physics Unit, A.O.U. Città della Salute e della Scienza di Torino, 10126 Turin, Italy; fgiglioli@cittadellasalute.to.it (F.R.G.); egallio@cittadellasalute.to.it (E.G.); 3 Radiation Oncology Unit, Department of Oncology, A.O.U. Città della Salute e della Scienza di Torino, 10126 Turin, Italy; ecuffini@cittadellasalute.to.it (E.M.C.); chia.cavallin@gmail.com (C.C.)

**Keywords:** total body irradiation, artificial intelligence, radiotherapy planning, decision support systems

## Abstract

**Background/Objectives**: Total body irradiation (TBI) is widely used in conditioning regimens before hematopoietic stem cell transplantation. In conventional opposed-field TBI, monitor unit (MU) calculation and lung shielding definition are often based on manual procedures that may introduce operator-dependent variability. This study aimed to develop machine learning (ML) models to support the prediction of these treatment parameters using routinely available clinical and imaging data. **Methods**: A retrospective analysis was performed on 80 patients treated with conventional opposed-field TBI. Clinical, geometric, and CT-derived variables were used to train regression models for MU prediction. Feature selection was performed using LASSO regression, followed by Ridge regression for final modeling. Lung shielding thickness prediction was developed using planning CT data from 66 patients through recursive feature elimination and Random Forest regression. Model performance was assessed using nested 5-fold cross-validation and mean absolute error (MAE). **Results**: The final Ridge model achieved an MAE of 74.0 ± 6.9 MU, improving compared with the full-feature model 115.6 ± 44.0 MU. The Random Forest benchmark achieved an MAE of 81.1 ± 10.3 MU. For lung shielding thickness prediction (6–9 mm), the Random Forest model achieved an MAE of 0.60 mm. Prediction uncertainties were consistent with clinically accepted in vivo dosimetric tolerances. **Conclusions**: ML-based models can support the estimation of key TBI treatment parameters, potentially improving workflow efficiency and reducing operator-dependent variability while complementing standard treatment planning and verification procedures.

## Simple Summary

Total body irradiation (TBI) is commonly used before hematopoietic stem cell transplantation. In conventional opposed-field TBI, treatment planning requires manual estimation of monitor units and lung shielding thickness, which may depend on operator experience. In this study, we developed machine learning models to predict these parameters using geometric measurements and CT-derived lung characteristics from previously treated patients. The models showed stable performance and prediction errors comparable to the variability observed in in vivo dosimetric verification. Rather than replacing standard treatment planning system calculations, these tools are intended to support clinical decision-making, potentially reducing planning time and improving consistency in conventional TBI workflows.

## 1. Introduction

Total body irradiation (TBI) remains, in some specific clinical conditions, a key component of conditioning regimens for hematopoietic stem cell transplantation in both adult and pediatric patients. Conventional TBI is most commonly delivered using pairs of opposed fields at extended source-to-surface distance, a technique that, despite its geometric simplicity, presents significant dosimetric and technical challenges due to large field sizes, patient heterogeneity, and the need to balance dose uniformity with organ-at-risk sparing, particularly for the lungs [1,2,3]. Pulmonary toxicity is one of the most relevant dose-limiting effects of TBI. Radiation-induced lung injury, including interstitial pneumonitis, has been shown to correlate with absorbed lung dose and treatment-related parameters such as beam weighting, patient separation, and shielding configuration [4,5,6]. For this reason, lung shielding is routinely implemented to reduce pulmonary dose while preserving adequate whole-body dose coverage. In recent years, intensity-modulated approaches to TBI and evolutionary approaches such as total marrow and lymphoid irradiation have gained increasing attention. Techniques based on multiple-isocenter Linac delivery or helical tomotherapy allow improved dose homogeneity and enhanced sparing of organs at risk, including the lungs [7,8,9,10]. These methods enable a high degree of modulation and personalization of treatment delivery. Nevertheless, despite these technological advances, conventional opposed-field TBI remains the most widely adopted technique in clinical practice worldwide. Its robustness, simplicity, limited sensitivity to setup uncertainties, and broad availability make it the standard approach in many institutions, particularly where access to advanced delivery systems is limited or where logistical efficiency is essential [11,12]. Within conventional TBI, the determination of monitor units (MUs) and compensator thickness remains a complex and largely experience-driven process. These parameters are typically derived through empirical rules and iterative manual calculations, resulting in workflows that are time-consuming and subject to inter-operator variability [13,14]. Although several studies have addressed lung shielding strategies and their dosimetric impact in TBI [4,15,16], limited attention has been given to predictive models capable of estimating treatment parameters directly from patient-specific data. In this context, machine learning (ML) has emerged as a promising tool for modeling complex relationships between patient characteristics and treatment parameters in radiation oncology [17,18,19]. Data-driven approaches have shown potential in improving consistency, reducing operator dependence, and supporting clinical decision-making. However, their application to conventional opposed-field TBI, particularly for the prediction of MUs and lung shielding parameters, remains largely unexplored. The development of reliable ML models critically depends on the availability of large, high-quality clinical datasets. Longitudinal cohorts spanning extended time periods, capture variability in patient anatomy, treatment setup, and clinical practice, thereby enhancing model robustness and generalizability. The aim of this study is to develop and validate a machine learning model based on a 14-year retrospective cohort of patients treated with myeloablative conventional opposed-field TBI, designed to predict monitor units and lung shielding parameters (Pb). This work seeks to provide a practical decision-support tool to reduce operator-dependent variability, streamline the planning workflow, and support effective lung sparing while maintaining adequate dose delivery.

## 2. Materials and Methods

### 2.1. Patient Cohort and Data Collection

This retrospective study included patients treated with conventional total body irradiation (TBI) at University Hospital “Città della Salute e della Scienza” of Turin between 2011 and 2024, covering a 14-year time period. Complete dosimetric data were available for 80 of 110 treated patients, with data missing for the remaining 30. All treatments were delivered using opposed photon fields at extended source-to-surface distance as part of the conditioning regimen for hematopoietic stem cell transplantation. All patients received a prescribed total dose of 12 Gy delivered using a hyper-fractionated regimen, with two fractions per day separated by at least 6 h over three treatment days, with respect to lung sparing, an acceptable maximum dose of 10 Gy out of the prescribed total dose of 12 Gy was defined. Clinical, anatomical, and treatment-related data were collected from the institutional radiotherapy database and treatment planning systems. Collected variables included patient demographics, treatment geometry parameters, monitor units (MUs), lung shielding parameters (Pb), and relevant setup-related measurements. Patients with incomplete dosimetric data or non-standard treatment configurations were excluded from the analysis. This retrospective study was based on fully anonymized technical and dosimetric data collected during routine clinical practice for quality assurance purposes. According to local institutional policy and applicable regulations, Ethics Committee approval and informed consent were not required.

### 2.2. Treatment Technique

All patients were treated using bilateral opposed fields delivered by two medical linear accelerators at extended source-to-surface distances (SSD) of 320 cm and 440 cm. A 6 MV photon beam was used with the gantry set at 270° and the collimator rotated to 45°. The prescribed dose was defined at the abdominal midline, corresponding to half the anteroposterior diameter at the level of the umbilicus, according to institutional protocols.

A maximum dose rate of 10 cGy/min at the patient level was applied. To improve surface dose and beam uniformity at the extended treatment distance, a plexiglass (PMMA) sheet was positioned in the beam path. Patient-specific shielding consisted of multiple 1 mm-thick lead layers individually shaped according to digitally reconstructed radiographs (DRRs) generated by the treatment planning system, allowing lung dose reduction while maintaining acceptable dose uniformity. Two treatment planning systems (TPSs) were used for monitor unit (MU) calculations: Oncentra MasterPlan (Nucletron B.V., later Elekta AB, Stockholm, Sweden; 2011–2017) and RayStation (RaySearch Laboratories AB, Stockholm, Sweden; 2017–2024), both using a collapsed cone convolution algorithm. MU calculations were performed on CT scans acquired in the supine position with the appropriate source-to-patient distance specified in the TPS. Patients were treated in a seated position using a dedicated immobilization frame with pelvic support and lateral stabilization bars to ensure setup reproducibility. Arm positioning followed institutional protocols, resting alongside the thighs.

Positioning was verified before each fraction using wall and laser references. Customized lung shields were mounted on the PMMA sheet to maintain geometric alignment with the lungs. Differences in patient thickness between the supine simulation and seated treatment position were compensated by measuring patient diameter in both positions and applying a corresponding virtual bolus on the CT dataset. In vivo dosimetry was performed using three diodes positioned at the umbilical level and behind the lung shielding to verify the delivered dose.

### 2.3. Feature Selection and Data Preprocessing

From the CT images, five anatomical distances were measured. In the sagittal plane, the distance from the cranial vertex to the coccyx and the anteroposterior thoracic diameter measured from the sternum to the dorsal spine were evaluated. In the coronal plane, the distance between the lung bases was measured. In the axial plane, the latero-lateral patient width was assessed at the level of the umbilicus and at the level of the femoral heads. For both the right and left lungs, lung volume and minimum, mean, and maximum Hounsfield Unit (HU) values were registered. In addition, the external body volume, including the skin, the prescribed dose to the lung, and the type of Treatment Planning System (TPS) used, were recorded. Before model development, all collected variables were reviewed for completeness and consistency. Continuous features were normalized, while categorical variables were encoded as appropriate. Outliers related to measurement errors or atypical treatment conditions were identified and excluded. The input features for the machine learning model included patient-specific anatomical parameters (body thickness, lung dimensions), treatment geometry variables, and setup-related quantities available at the time of treatment planning (Table 1). The target variables were monitor units (MUs) and lung shielding thickness (Pb), expressed in mm. Feature selection was performed using LASSO regression to reduce redundancy and improve model interpretability.

### 2.4. Machine Learning Model for Monitor Unit Prediction

A retrospective dataset of 80 patients was used to develop a supervised regression model for monitor unit (MU) prediction. Twenty variables were available for each patient, including treatment machine parameters, geometric measurements, lung volumes and density descriptors, external contour volume, and delivered MUs. The linear accelerator (Linac) identifier was included as a categorical variable to account for machine-related variability, while MUs were treated as a continuous target variable. Missing values were handled using median imputation and continuous variables were standardized to zero mean and unit variance. All preprocessing steps were implemented within model pipelines to prevent information leakage during validation. In particular, imputation, normalization, and feature selection procedures were performed independently within each training fold of the nested cross-validation process, and the corresponding validation/test folds were never used during preprocessing or model optimization stages. Model development and evaluation were performed using nested cross-validation, with a 5-fold outer loop for performance estimation and a 5-fold inner loop for hyperparameter optimization through grid search. Recursive Feature Elimination with Cross-Validation (RFECV) and LASSO-based feature selection were implemented exclusively within the inner cross-validation loop and recalculated at each iteration using only the corresponding training subset, thereby ensuring a fully leakage-free evaluation framework [20]. Model performance was assessed using the mean absolute error (MAE), selected for its direct clinical interpretability. Feature selection was performed using LASSO regression (L1 regularization) [21], which enables simultaneous coefficient shrinkage and variable selection. The regularization parameter was optimized within the nested cross-validation framework. Predictors associated with non-zero coefficients were retained, resulting in a subset of five variables. A Ridge regression model (L2 regularization) [22] was then trained using the selected predictors to mitigate residual multicollinearity and stabilize coefficient estimates while preserving interpretability. For comparison, a Random Forest regressor [23] with constrained model complexity was also evaluated as a non-linear reference model. Finally, the coefficients of the Ridge model were analyzed to assess the direction and clinical plausibility of predictor–MU relationships. All analyses were performed in Python using the scikit-learn library [24].

### 2.5. Machine Learning Model for Lung Shielding Thickness (Pb) Prediction

Planning CT data from 66 patients (82.5%) were used to develop a supervised regression model to predict lead (Pb) lung shielding thickness. Fourteen patients were excluded due to stricter lung dose constraints (≤8 Gy), applied in selected cases to mitigate pulmonary toxicity, particularly when patient- or treatment-related factors warranted a more conservative approach. Sixteen CT-derived quantitative features were extracted for each patient, including geometric parameters, lung volumes, and lung density descriptors expressed in Hounsfield Units (HU). The Linac identifier was included as a categorical variable to account for machine-related variability. Numerical features were standardized using z-score normalization and the Linac variable was encoded using one-hot encoding.

No missing values were present. Feature selection was performed using recursive feature elimination with cross-validation (RFECV) applied exclusively within the training data of each cross-validation split to avoid information leakage. A Ridge regression estimator was used within RFECV due to its stability in small datasets and ability to handle correlated predictors. MAE was used as the optimization metric, with a minimum of five features enforced. Across folds, a stable subset of five CT-derived features was consistently selected: patient diameter and the minimum and maximum HU values of both lungs. The Linac identifier was retained as an additional covariate. A Random Forest regressor was used as the final predictive model due to its ability to capture non-linear relationships and its robustness with limited data. Model complexity was constrained to reduce overfitting, and hyperparameters were optimized within the inner cross-validation loop. Model performance was evaluated using nested 5-fold cross-validation with MAE as the primary metric, and RMSE and R^2^ reported for completeness. A final model was then trained on the full dataset using the selected features.

### 2.6. In Vivo Dosimetry and Data Analysis

In vivo dosimetry was performed to independently assess dose delivery accuracy in patients treated with conventional opposed-field TBI. Measurements were acquired during treatment using EDP-10 silicon diodes (Scanditronix Wellhöfer, later IBA Dosimetry, Schwarzenbruck, Germany) according to institutional quality assurance procedures.

Dosimeters were positioned on the patient surface at two clinically relevant locations: along the patient central axis and posterior to the lungs, in order to evaluate both global dose delivery and the effectiveness of lung shielding. Placement was performed at predefined anatomical landmarks to ensure reproducibility across patients and fractions.

Diode placement and in vivo dose recording were performed during the first two of the six scheduled treatment sessions. In vivo dosimetry results were recorded as two measurements per treatment session, corresponding to the anteroposterior (AP) and posteroanterior (PA) beam directions, with the PA measurement obtained after a 180° patient rotation.

For each beam, the dose at half patient diameter along the central axis, derived according to the adopted method [25], and the dose at beam exit over the lung region were recorded and compared with treatment planning values. Accordingly, four measurements were available for each patient: two AP measurements acquired during the first treatment session and two PA measurements acquired during the second session. Diode calibration was performed according to the methodology described in the aforementioned publication. Each diode was assigned individual calibration factors for entrance dose, exit dose, and measurements acquired behind the lead shield.

The dose measured by diodes at midline (corresponding to half the patient diameter) was compared with the prescribed dose, while the dose measured behind the lead shielding was compared with the dose calculated by the treatment planning system (TPS); dosimetric agreement was quantified as the relative dose difference (%) between measured and expected values. In vivo dosimetry results were analyzed across the patient cohort using descriptive statistics. Mean and standard deviation of relative dose differences were reported separately for central-axis measurements and post-lung measurements.

## 3. Results

### 3.1. Patient Characteristics

In the selected patient cohort, the mean age was 35.9 ± 12.4 years (range: 13–63 years).

Treatments were delivered using different linear accelerators, with two source-to-surface distance (SSD) configurations employed: 340 cm (Linac 1) and 440 cm (Linac 2). The mean antero-posterior patient diameter was 25.1 ± 3.7 cm, while the mean CT-based diameter was 20.9 ± 3.8 cm, consistently smaller than the external measurement. Five anatomical distances were extracted from CT images, describing patient size and thoracic geometry in the sagittal, coronal, and axial planes, and were used as geometric descriptors of inter-patient variability. The right lung volume averaged 1489 ± 452 cm^3^, whereas the left lung volume averaged 1347 ± 1037 cm^3^, with a larger dispersion observed for the left lung due to extreme values. For the right lung, minimum, mean, and maximum Hounsfield Unit (HU) values were −931 ± 61 HU, −64 ± 160 HU, and 129 ± 127 HU, respectively.

Corresponding values for the left lung were −910 ± 63 HU, −63.4 ± 152 HU, and 104 ± 97 HU.

The mean external body volume, including the skin, was 50,320 ± 11,952 cm^3^ (range: 28,596–78,421 cm^3^). The monitor units delivered (MU) showed a clear dependence on the SSD configuration. For treatments delivered at 340 cm SSD (*n* = 31), the mean MU was 2578 ± 93 MU, with values ranging from 2386 MU to 2742 MU. For treatments delivered at 440 cm SSD (*n* = 49), the mean MU was 4489 ± 26 MU, with a wider range spanning from 4024 MU to 5153 MU. Regarding the Pb thickness, a total of 66 observations were analyzed, referring to two linear accelerators (Linac 3 and Linac 4). Linac 3 accounted for 24 measurements (36.4%), while Linac 4 accounted for 42 measurements (63.6%). The lead (Pb) thickness ranged from 6 to 9 mm across the entire dataset, with an overall median thickness of 8 mm for both Linacs. Overall, the distribution of Pb thickness values was comparable between the two Linacs, with no substantial differences observed in central tendency or variability.

### 3.2. Model Performance and Feature Selection for MU Prediction

**Figure 1:**
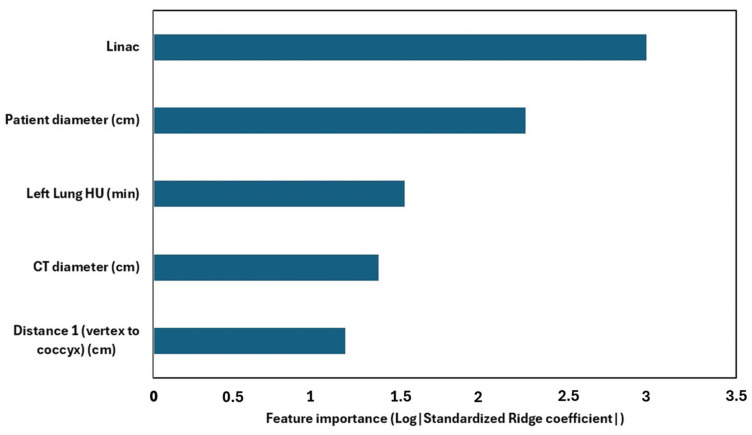
Feature importance derived from the standardized coefficients of the Ridge regression model. The Linac identifier emerged as the dominant predictor, reflecting differences in source-to-patient distance between machines. Among patient-related variables, patient diameter showed the strongest contribution, followed by anatomical and imaging-related parameters, which exhibited a comparatively lower influence on the model. A logarithmic scale was applied to the *x*-axis to improve the visualization of lower-magnitude feature contributions in the presence of the dominant Linac-related coefficient.

Model performance results are summarized in Table 2. The initial Ridge regression model trained on the full set of input variables showed limited predictive accuracy, with a mean absolute error (MAE) of 115.6 ± 44.0 MU. Feature selection using LASSO regression led to a substantial improvement in predictive performance while reducing model complexity. The LASSO model selected five predictors (Linac, patient diameter, left lung HU minimum, CT diameter, distance vertex to the coccyx, Figure 1) achieving a mean MAE of 84.1 ± 12.7 MU. The Random Forest regressor, evaluated as a non-linear benchmark using all input features, achieved a mean MAE of 81.1 ± 10.3 MU.

#### 3.2.1. Final Ridge Model Performance

The best overall performance was obtained by the Ridge regression model trained on the five features selected by LASSO, which achieved a mean MAE of 74.0 ± 6.9 MU across the outer folds of the nested cross-validation (Table 2), indicating improved accuracy and reduced variability.

The final Ridge regression model trained exclusively on the LASSO-selected features demonstrated the lowest prediction error and the highest stability among all evaluated models. The optimized regularization parameter was consistently low across cross-validation folds, indicating that the selected predictors retained most of the relevant information with limited residual multicollinearity. Compared with the non-linear Random Forest benchmark, the final Ridge model achieved superior predictive performance, with a reduction in MAE of approximately 7 MU. While the Random Forest model provided competitive accuracy, its performance remained inferior to that of the final Ridge model and was not considered suitable for clinical interpretability.

#### 3.2.2. Summary of Results

Overall, the combination of LASSO-based feature selection followed by Ridge regression provided the most favorable balance between predictive accuracy, robustness, and interpretability. This approach outperformed both the full-feature linear model and the non-linear benchmark while relying on a reduced and clinically meaningful subset of predictors.

### 3.3. Pb Shielding Thickness Prediction

#### 3.3.1. Dataset Characteristics

The final dataset included 66 patients. Lead shielding thickness ranged from 6 to 9 mm. In both machines, the most frequently used thicknesses were 7 mm and 8 mm, accounting for most cases. Linac 3 showed a higher relative use of 8 mm shielding (45.8%), whereas Linac 4 more frequently employed 7 mm shielding (40.5%). However, the overall distribution of shielding thickness did not differ significantly between the two Linacs. A chi-square test of independence showed no statistically significant association between Linac type and lead thickness selection (χ^2^ = 1.42, *p* = 0.70), indicating comparable clinical practice across the two machines.

#### 3.3.2. Feature Selection Results

Feature selection performed within the nested cross-validation framework consistently identified a limited subset of CT-derived predictors. Patient diameter was selected in all outer folds, indicating high stability. In addition, extreme lung density values expressed in Hounsfield Units (HU) were frequently retained, specifically the minimum and maximum HU values of the right lung and left lung volume. Volume of the lungs and mean HU values were selected less consistently and were excluded from the final feature set. Based on feature stability and physical interpretability, a fixed subset of five CT-derived features was selected for the final model: patient diameter; minimum and maximum HU of the right lung; and minimum and maximum HU of the left lung volume. The Linac identifier was retained as a categorical covariate to account for potential machine-related variability.

#### 3.3.3. Nested Cross-Validation Performance

Model performance was evaluated using a fully nested 5-fold cross-validation framework. The mean absolute error (MAE) across outer folds was 0.60 mm, with values ranging from 0.47 to 0.73 mm. The root mean squared error (RMSE) was 0.75 mm, while the coefficient of determination (R^2^) fluctuated around zero. Despite the limited dynamic range of the target variable and the small sample size, the model achieved low absolute prediction error with stable performance across all outer folds, with no evidence of systematic overfitting.

#### 3.3.4. Model Robustness and Error Analysis

Prediction errors were primarily observed near adjacent Pb thickness values (e.g., 7 mm vs. 8 mm). No large deviations were observed, with no predictions differing by more than 2 mm from the true value. Performance remained consistent across all outer folds, supporting the robustness of the modeling approach.

#### 3.3.5. Final Model Training

**Figure 2:**
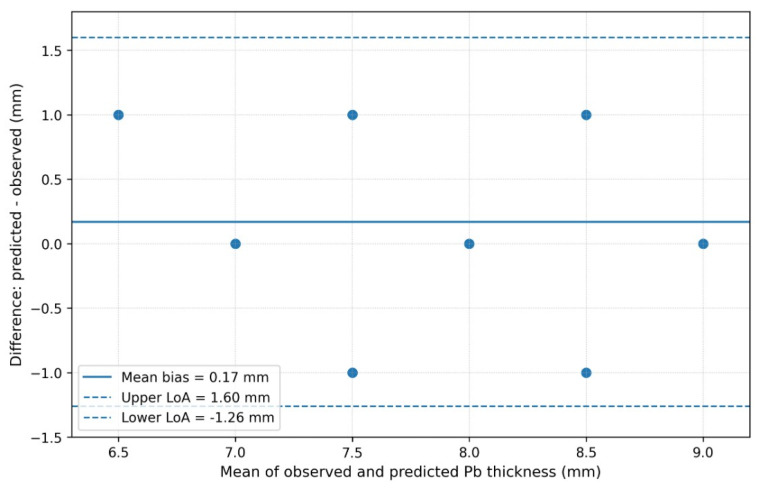
Bland–Altman plot comparing observed and machine learning–predicted Pb shielding thickness values. The solid horizontal line represents the mean bias between predicted and observed values (0.17 mm), while the dashed lines indicate the 95% limits of agreement (−1.26 mm to 1.60 mm). Most prediction differences remained within ±1 mm, with no evident systematic trend across the investigated range of Pb thicknesses, supporting the clinical acceptability of the proposed model.

Following nested cross-validation, the Random Forest model was retrained on the full dataset using the selected CT-derived features and the Linac categorical variable (Figure 2). This model represents the operational configuration intended for downstream analyses and potential clinical decision support.

### 3.4. In Vivo Dosimetry

In vivo dosimetry was performed along the central axis (CAX) and posterior to the lungs to evaluate dose delivery accuracy in patients treated with conventional opposed-field TBI. A total of 80 paired measurements were available for analysis. For central-axis measurements, the mean dose deviation was 0.6% ± 5.4% for the AP direction and 1.3% ± 4.5% for the PA direction. The variability of central-axis in vivo dosimetry deviations, expressed as interquartile range (IQR), was 8.0% for AP measurements and 6.0% for PA measurements. At the lung exit region the mean deviation was −1.1% ± 8.8% for AP beams and 4.7% ± 7.5% for PA beams. The IQR was 10.3% for AP measurements and 7.7% for PA measurements, confirming the relatively broad dispersion of lung in vivo dosimetry deviations in anatomically heterogeneous regions during conventional TBI treatments. A statistically significant difference between AP and PA measurements was observed at the lung exit region (paired *t*-test, *p* < 0.001), whereas no significant difference was found along the central axis. During in vivo measurements, corrective actions were implemented when relevant deviations from planned doses were observed. For central-axis measurements, monitor units were scaled or adjusted in the presence of deviations exceeding 5%.

Conversely, modifications of lead shielding thickness were considered only for larger deviations (≥7–10%), as diode readings at lung exit were more susceptible to setup-related uncertainties, tissue heterogeneities and because the presence of lead could potentially introduce energy-response biases, given the difference between the beam spectrum at the measurement point and the calibration conditions of the detectors.

## 4. Discussion

In recent years, artificial intelligence (AI) has had a major impact on radiation oncology, with applications spanning image segmentation, treatment planning, outcome prediction, toxicity assessment, and quality assurance [26]. Although deep learning models achieve high performance, they require large annotated datasets, which are often difficult to obtain in medicine and oncology due to the rarity of several diseases and clinical subgroups. Consequently, classical machine learning methods such as support vector machines, random forests, and gradient boosting remain highly valuable in limited-data settings. These approaches have been successfully used to integrate radiomic, clinical, and dosimetric data for outcome prediction and toxicity modelling in personalized radiation therapy [27,28,29].

In this study, machine learning models were developed to support the prediction of monitor units (MUs) and lung shielding thickness (Pb) in patients treated with conventional opposed-field total body irradiation (TBI). Using retrospective data collected over a 15-year period, the proposed approaches demonstrated stable performance, clinical interpretability, and consistency with in vivo dosimetric measurements. For MU prediction, the combination of LASSO-based feature selection and Ridge regression provided the best balance between accuracy and robustness, achieving a mean absolute error of approximately 74 MU. Feature selection substantially improved model performance compared with a full-feature linear model, highlighting the importance of dimensionality reduction when modeling heterogeneous clinical and geometric variables. The variables selected by the model, including CT-derived diameter, vertex-to-coccyx distance, and extreme lung HU values, may reflect clinically relevant anatomical and dosimetric characteristics influencing dose attenuation and irradiation geometry during extended SSD TBI treatments.

In particular, CT diameter and anatomical longitudinal distances can be considered surrogate descriptors of patient body size and effective beam path length, while minimum and maximum lung HU values may indirectly characterize lung tissue heterogeneity and density distribution, potentially affecting attenuation conditions and shielding requirements. Although some of these predictors may not have a direct standalone dosimetric interpretation, their selection likely reflects the ability of the model to capture combined geometric and anatomical information relevant to treatment parameter estimation. Although a Random Forest model showed competitive accuracy, the final Ridge model offered superior interpretability, which is particularly relevant in the context of TBI, where treatment parameter estimation is traditionally grounded in physical reasoning. The preference for relatively simple and interpretable models, such as Ridge and LASSO regression, over more complex deep learning architectures [30] was also motivated by the limited dataset size and the structured nature of the available variables. In this setting, regularized linear models provide a favorable balance between predictive performance, robustness, and interpretability, while reducing the risk of overfitting typically associated with highly parameterized models in small datasets. This choice is consistent with current recommendations promoting interpretable machine learning approaches in healthcare applications, where transparency and clinical reliability are essential for safe implementation. For lung shielding thickness prediction, the Random Forest model achieved sub-millimeter accuracy, despite the limited dynamic range of the target variable. Feature selection consistently identified a small subset of CT-derived predictors, including patient diameter and extreme lung density values, suggesting that both patient geometry and lung heterogeneity play a key role in determining shielding requirements. Prediction errors were primarily confined to adjacent thickness values, with no large deviations observed.

Nevertheless, some limitations of the present study should be acknowledged. The relatively limited sample size may reduce statistical power and increase the risk of feature instability and model overfitting, despite the adoption of nested cross-validation to mitigate optimistic bias and provide a more robust estimate of model performance. In addition, the absence of an independent external validation cohort limits the generalizability of the proposed models across different institutions, treatment workflows, and patient populations [31]. Future multicenter studies based on larger and more heterogeneous datasets will therefore be necessary to externally validate and further refine the proposed framework. An additional limitation is that the retrospective dataset included treatment plans generated using two different TPS platforms over the study period. However, both systems employed Type B collapsed cone convolution algorithms, and internal comparative recalculations performed during the TPS transition did not reveal significant differences in calculated MUs or lung shielding thickness estimations; therefore, a substantial impact on model performance is not expected. Finally, the limited range of Pb shielding thickness values included in the dataset may restrict model generalizability to institutions adopting different lung shielding protocols.

Although in vivo dosimetry was not used for model training, the prediction uncertainty of the MU and lead thickness models was comparable to the intrinsic variability of in vivo verification. In particular, central-axis in vivo deviations showed a standard deviation of approximately 5% (AP: 0.6% ± 5.4%; PA: 1.3% ± 4.5%), while lung-exit measurements exhibited a larger dispersion of approximately 8% (AP: −1.1% ± 8.8%; PA: 4.7% ± 7.5%). The cross-validated error of the MU model, corresponding to about 74 MU over typical values of 2578–4489 MU (≤2.8%), remained below the institutional clinical intervention threshold of 5% adopted for in vivo diode dosimetry within the TBI program, and the lead thickness model, with an error of 0.6–0.75 mm relative to a nominal thickness of 8 mm (≈8%), was consistent with the clinically adopted tolerance criteria, where deviations greater than approximately 7–10% generally resulted in increasing or decreasing the shielding thickness by 1 mm of lead. Therefore, the prediction uncertainty falls within the uncertainty envelope of conventional TBI delivery and in vivo verification, particularly when considering the higher variability observed in anatomically heterogeneous regions such as the lungs. In this context, the proposed ML models may support the clinical workflow by providing rapid preliminary estimates of treatment parameters prior to standard TPS verification and routine in vivo dosimetry checks. Despite the increasing adoption of intensity-modulated approaches for TBI, conventional opposed-field techniques remain widely used in clinical practice.

In this setting, the proposed models are not intended to replace established calculation or verification procedures but rather to act as decision-support tools for rapid preliminary estimation of treatment parameters within the conventional TBI planning workflow.

Nevertheless, despite the limitations discussed above, the results demonstrate the feasibility of using machine learning to support treatment parameter estimation in conventional TBI while preserving established clinical workflows. Future validation on independent multicenter cohorts may eventually further support the broader applicability of the proposed framework.

## 5. Conclusions

This study demonstrates the feasibility of using machine learning models to support the estimation of monitor units and lung shielding thickness in conventional opposed-field total body irradiation. The proposed models achieved clinically acceptable prediction accuracy while maintaining interpretability and consistency with in vivo dosimetric verification. Rather than replacing conventional TPS-based calculations, these approaches are intended to complement standard planning procedures as decision-support tools. External validation on independent datasets is a potential future step to confirm generalizability.

## Figures and Tables

**Table 1:** Summary of all candidate input variables extracted from the two datasets used for development of the MU and Pb thickness prediction models. The table reports the definition and unit of each variable, the model in which it was evaluated, and whether the variable was retained after the feature selection process for the final predictive models.

Variable	Definition	Unit	Model
Linac	Linear accelerator energy/configuration identifier	categorical	MU + Pb
SSD	Source-to-surface distance	mm	MU
TPS	Treatment planning system identifier	categorical	MU
Patient diameter	Maximum patient lateral diameter	cm	MU + Pb
CT diameter	Diameter measured on CT images	cm	MU
Distance 1 (vertex to coccyx)	Cranio-caudal patient length from vertex to coccyx	cm	MU
Distance 2 (max AP thorax)	Maximum anteroposterior thoracic diameter	cm	MU
Distance 3 (max LL lung base)	Maximum laterolateral diameter at lung bases	cm	MU
Distance 4 (max LL umbilicus)	Maximum laterolateral diameter at umbilical level	cm	MU
Distance 5 (max LL pelvis)	Maximum laterolateral pelvic diameter	cm	MU
Right lung volume	Right lung segmented volume	cm^3^	Pb
Right lung HU min	Minimum Hounsfield Unit in right lung	HU	Pb
Right lung HU average	Mean Hounsfield Unit in right lung	HU	Pb
Right lung HU max	Maximum Hounsfield Unit in right lung	HU	Pb
Left lung volume	Left lung segmented volume	cm^3^	MU + Pb
Left lung HU min	Minimum Hounsfield Unit in left lung	HU	MU + Pb
Left lung HU average	Mean Hounsfield Unit in left lung	HU	Pb
Left lung HU max	Maximum Hounsfield Unit in left lung	HU	Pb
Total lung volume	Sum of left and right lung volumes	cm^3^	Pb
Total lung HU min	Minimum Hounsfield Unit across lungs	HU	Pb
Total lung HU average	Mean Hounsfield Unit across lungs	HU	Pb
Total lung HU max	Maximum Hounsfield Unit across lungs	HU	Pb
External body volume	External patient contour volume	cm^3^	MU + Pb
Monitor Units (MU)	Delivered monitor units	MU	MU (target variable)/Pb (input)
Pb thickness	Lead compensator thickness	mm	Pb (target variable)

**Table 2:** Performance of different predictive models for monitor unit (MU) estimation. For each model, the feature selection strategy, number of selected features, and mean absolute error (MAE) expressed in MU (mean ± standard deviation) are reported.

Model	Feature Selection	No. of Features	MAE (MU), Mean ± SD
Ridge (full model)	None	20	115.6 ± 44.0
LASSO	Embedded (L1)	5–6	84.1 ± 12.7
Random Forest	None	20	81.1 ± 10.3
Ridge (selected features)	LASSO-based	5	74.0 ± 6.9

## Data Availability

The data presented in this study and the analysis code are available from the corresponding author on reasonable request.
